# Agro-active endo-therapy treated *Xylella fastidiosa* subsp. *pauca*-infected olive trees assessed by the first ^1^H-NMR-based metabolomic study

**DOI:** 10.1038/s41598-022-09687-8

**Published:** 2022-04-08

**Authors:** Chiara Roberta Girelli, Mudassar Hussain, Dimitri Verweire, Michael C. Oehl, Josep Massana-Codina, Maier S. Avendaño, Danilo Migoni, Marco Scortichini, Francesco Paolo Fanizzi

**Affiliations:** 1 Department of Biological and Environmental Sciences and Technologies, University of Salento, 73100 Lecce, Italy; 2 Invaio Sciences, Cambridge, MA 02138 USA; 3 Research Centre for Olive, Fruit and Citrus Crops, Council for Agricultural Research and Economics (CREA), 00134 Rome, Italy

**Keywords:** Metabolomics, NMR spectroscopy, Plant sciences, Biotic

## Abstract

*Xylella fastidiosa* is a xylem-limited bacterium causing a range of economically important plant diseases in hundreds of crops. Over the last decade, a severe threat due to Olive Quick Decline Syndrome (OQDS), caused by *Xylella fastidiosa* subspecies *pauca,* affected the Salento olive groves (Apulia, South-East Italy). Very few phyto-therapeutics, including a Zn/Cu citric acid biocomplex foliar treatment, were evaluated to mitigate this disease. However, the traditional foliar applications result in the agro-actives reaching only partially their target. Therefore the development of novel endo-therapeutic systems was suggested. Metabolite fingerprinting is a powerful method for monitoring both, disease progression and treatment effects on the plant metabolism, allowing biomarkers detection. We performed, for the first time, short-term monitoring of metabolic pathways reprogramming for infected Ogliarola salentina and Cima di Melfi olive trees after precision intravascular biocomplex delivery using a novel injection system. Upon endo therapy, we observed specific variations in the leaf content of some metabolites. In particular, the ^1^H NMR-based metabolomics approach showed, after the injection, a significant decrease of both the disease biomarker quinic acid and mannitol with simultaneous increase of polyphenols and oleuropein related compounds in the leaf’s extracts. This combined metabolomics/endo-therapeutic methodology provided useful information in the comprehension of plant physiology for future applications in OQDS control.

## Introduction

Olive (*Olea europea* subsp. *europea*) trees are evergreen plants that are usually grown in the Mediterranean regions for oil production. This area is characterized by summers with high temperatures, high levels of sunlight, and high humidity conditions that are suitable for olive cultivation^1^. In Italy, the central-southern territory is largely dedicated to olive cultivation. Apulia (South-East Italy) is the region with the largest production and olive oil is among the most important sources of income for the local economy^2^. In addition, century-old olive trees represent a unique landscape part of the cultural heritage of the region with high touristic value. Over the last decade, the olive groves of Salento (South Apulia), are experiencing a very severe threat due to a disease named Olive Quick Decline Syndrome (OQDS). This disease is caused by the quarantine bacterial pathogen *Xylella fastidiosa* subspecies *pauca* which is originated and introduced from Central America^3^. *X. fastidiosa* is a xylem-limited bacterium that causes a range of economically important plant diseases in hundreds of crops, including grapevine, almonds, and olive trees. It is well known especially in the Americas, where it is an endemic pathogen^4^. OQDS can be revealed by a-specific symptoms such as severe wilting in leaves, twigs, and branches. Often the disease progression results in plant death. The European quarantine legislation (EQL) is trying to limit the spread of pathogens across the countries. Accordingly, a specific Apulia mandatory legislation has divided the southeast part of the region into three areas for having better control of the disease. The infected area where the disease is widespread and cannot be eliminated, the “containment” area adjacent to the infected one, where uprooting of the infected plants must be performed upon a positive detection, and the “buffer” area, where also all surrounding trees in a radius of 50 m from the infected tree must be uprooted^5^. Currently, several efforts are being made to develop control measures for OQDS mitigation^6^. Among the disease control attempts, usage of commercially available DENTAMET, a biocomplex containing zinc (4%), copper (2%), and citric acid, has been assessed as a foliar treatment able to reduce the *X. fastidiosa* subsp. *pauca* cell concentration in olive trees^7^. Treated plants continue to vegetate and keep their productivity^8,9^. In addition to assessing disease control by quantitative PCR analyses, upon the foliar treatments with the biocomplex, OQDS biomarkers and metabolic pathways re-programming have been investigated by ^1^H NMR-based metabolomics^8^. This methodology, based on spectroscopic analyses of xylematic extracts obtained from leaves, enabled the identification of specific disease-related metabolites. Notably, besides the comparison of the pathogen-induced metabolic differences, concerning the healthy plants, the metabolomic approach also allowed the post-treatment follow-up for the infected trees at a molecular level. Among the analytical techniques applied currently in the agri-food metabolomic field, Nuclear Magnetic Resonance (NMR) has proved to be very efficient for the characterization of specific substrates with simultaneous metabolites detection without prior components separation. In our previous study, we used non-targeted ^1^H-NMR fingerprinting, in combination with unsupervised and supervised pattern recognition techniques, for the response characterization, of naturally-infected olive trees, to the DENTAMET foliar treatments^2,10,11^. We reported that the effect of the treatment resulted in the specific involvement of xylematic polyphenols and carbohydrates for the studied cultivars (including both *Xylella* susceptible and resistant cultivars).

In the present study, we followed a combined method to investigate the effects of DENTAMET on endo-therapy treated plants. Trunk injection is a particular technique used to introduce agrochemical compounds into trees^12^. Since the traditional foliar application of agro-actives results in only 70–80% reaching their target in the plant, the development of a novel endo-therapeutic precision systems has been suggested also in this specific case. The endo-therapy can be considered as an environmentally friendly way to control bacterial diseases as it safely and precisely delivers the biological control agents directly into the plant body^13^. In urban environments, direct injection in plants of protection chemicals is a superior method that promises to manage pests and pathogens at the finest level. Endo-therapy has several advantages, such as: high efficiency or pathogen control, significantly reduced environmental pollution, significantly reduced risk to workers. It becomes the only choice when soil or foliar applications are ineffective^13^ and enables the possible use of biological actives that are expensive to produce or unstable in the environment. The use of traditional trunk injection has been limited due to several important constraints, the main one being the significant plant damage created after trunk drilling (4 to 12 mm, 5 to 120 mm depth). Additionally, in the traditional injection, the formulated active ingredient is delivered to the heartwood of the trunk, which presumably leads to a slower distribution throughout the plant. Indeed, in contrast to the active xylem, which transports water and nutrients throughout the plant^14^, the heartwood has a limited physiological role, and the sap flux decreases significantly towards the heartwood boundary. In the present study, we used a combined method to investigate the effects of DENTAMET on plants treated by a novel endo-therapy system. Foliar spray of agrochemicals is suitable to control pathogens living on the leaf, for invaders like *X. fastidiosa* that attack the xylem, the agrochemicals are expected to be more efficiently injected directly in xylem vessels. The current study reports on the metabolomic investigations performed by Nuclear Magnetic Resonance spectroscopy on leaf extracts after endo-therapeutic treatment using a novel precision injection system (referred to herein as “the TIPS System” available from Invaio Sciences) of OQDS infected olive trees. As far as we know this is the first report on a metabolomic study of endo-therapy treated *X. fastidiosa* infected olive trees.

## Results

### ^1^H-NMR characterization of olive leaf aqueous extract

**Figure 1:**
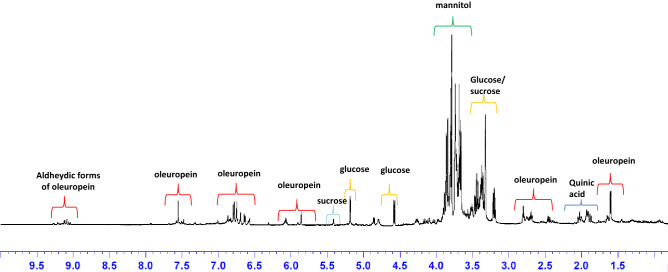
Typical aqueous ^1^H NMR zgcppr spectrum of olive leaf extract. Main metabolites are indicated.

The 84 leaves samples used for extract preparation were selectively collected at different times, before and after the endo-therapy treatment from four *X. fastidiosa* infected trees (two Ogliarola salentina and two Cima di Melfi cultivars) in the Carovigno municipality (Brindisi province, Apulia region). Every considered time point consisted of three samples for each tree (six for Ogliarola salentina and six for Cima di Melfi). Samples were collected before treatment and 0.25, 1, 2, 4, 7, and 15 days after the Zn/Cu citric acid biocomplex (DENTAMET) injection. The typical aqueous ^1^H NMR spectrum of olive leaf extract is reported in Fig. 1. Among the detectable compounds, oleuropein results as one of the main olive leaves’ components^15^ with the signals related to its functional groups indicated in Fig. 1. Moreover, signals of sugars such as sucrose, glucose, and mannitol are also shown. The signals of quinic acid were also identified and are pointed out in the spectrum.

### Multivariate statistical analysis

**Figure 2:**
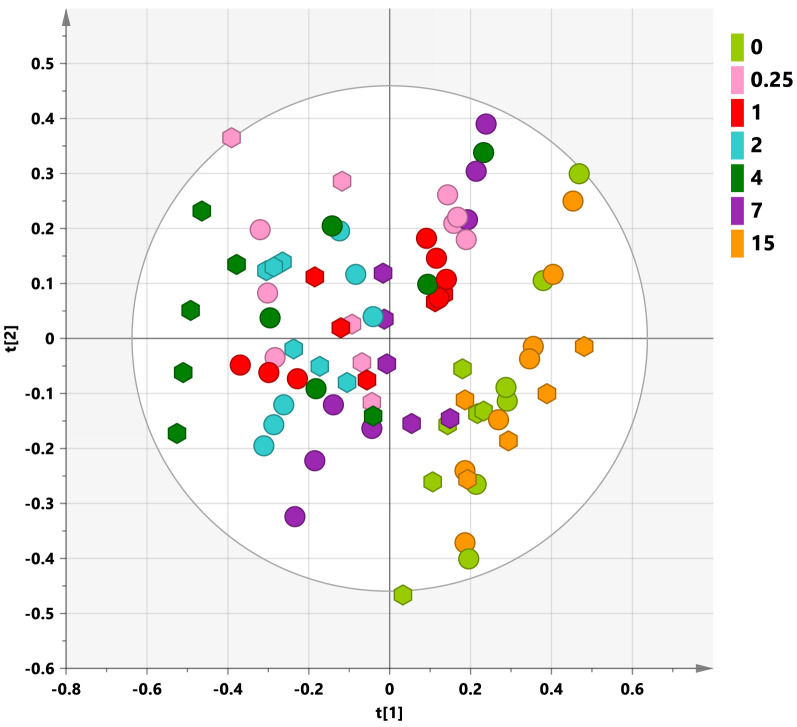
PCA t[1][2] scores plot (five components give R2X = 0.832, Q2 = 0.757) for leaf samples from Ogliarola salentina (circles) and Cima di Melfi (hexagons) cultivars tree at day 0 (before injection) and 0.25, 1, 2, 4, 7 and 15 days after injection. Sample symbols are colored according to different days of the treatment.

The unsupervised PCA analysis, performed using the buckets row reduced spectra, on the whole, 84 samples data set, showed the unsupervised grouping of the leaf extracts (Fig. 2). By visual inspection of the scores plot, the separation of the samples along the first component could be observed. In particular, samples from day 0 and day 15 appeared to group together at positive values of the first principal component. On the other hand, all other samples from days 0.25, 1, 2, 4, and 7 resulted distributed in the scores plot, with not marked clustering, from positive (0.3) to negative (− 0.6) values of the t1 component. Interestingly, these features are characteristic for both Ogliarola salentina and Cima di Melfi cultivars also when considered separately (Supplementary Figs. S1 and S2). This preliminary analysis also suggests the occurrence, for both cultivars, of similar treatment related metabolic profiles. In particular olive leaves extracts metabolites appear similar for day 0 and day 15 samples whereas such specific comparable metabolic profiles (day 0 and day 15) clearly result different from those observed at times 0.25, 1, 2, 4, and 7.

To further investigate the grouping trend observed in the PCA analysis, a series of pairwise OPLS DA analyses (Supplementary Fig. S3) was used to compare time 0 (pre-treatment sample) to each of the remaining samples taken at different days post treatment. The predictive ability of the six resulting models was then compared by evaluating the values of their Q2 parameters (Table 1). The Q2 values observed for all six models described a satisfactory predictive ability indicating effective discrimination between the pre-treatment and post-treatment samples. Interestingly, the lowest Q2 value (0.441) and consequent lowest discrimination ability of the model, was obtained for the pair related to time 0 (pre-treatment) and 15 days after treatment. This confirmed the previous observation based on simple PCA data analysis. Moreover, the highest value of Q2 was obtained for the model comparing 2 days after the injection with time 0 samples (pre-treatment), indicating the occurrence, at that specific time, of the highest observed discrimination within the six evaluated pairwise OPLS-DA comparisons. Again, the observed trend was the same also considering separately the two different cultivars (Supplementary Figs. S4 and S5, Supplementary Table S1).

**Table 1:** Statistical parameters of supervised OPLS-DA models comparing time 0 with each of the considered times. R2X and R2Y indicate the fraction of variance of the X and Y matrix, respectively. Q2 is a goodness of prediction parameter representing the portion of the variance in the data predictable by the model.

OPLS-DA (1 + 1 + 0)	R2X	R2Y	Q2
Days 0 vs 0.25	0.494	0.909	0.808
Days 0 vs 1	0.543	0.894	0.806
Days 0 vs 2	0.62	0.946	0.916
Days 0 vs 4	0.633	0.876	0.85
Days 0 vs 7	0.283	0.936	0.862
Days 0 vs 15	0.508	0.804	0.694

OPLS-DA analyses (Supplementary Figs. S3, S4, and S5), comparing the metabolic profiles at different days after the injection with respect to day 0, showed, besides a marked separation of the considered classes along with the predictive component (t1) also a clear intraclass variation along the orthogonal (to1) component. A further analysis of the OPLS-DA score plots indicates that the observed intra-class variation stems from discrimination between cultivars (Supplementary Fig. S3) and even between the two studied trees for each cultivar (Supplementary Figs. S4 and S5). Interestingly also the observed intraclass separations between cultivars (Supplementary Fig. S3) and trees within the same cultivar (Supplementary Figs. S4 and S5) appears to be time dependent indicating a maximum differentiation occurring in both cases at 1–2 days after the treatment and a further smoothing at the end of the monitoring (15 days). Analogous effect of improved cultivar differentiation occurring, after foliar treatment with DENTAMET of *X. fastidiosa* infected plants was previously observed by ^1^H NMR data supported multivariate analysis studies^10^. Moreover, the observed discrimination between the two trees for each cultivar clearly indicates also a possible specific response according to the plant physiological conditions (Table 3). As observed by pairwise OPLS-DA, comparing the two cultivars (1 day after the endo therapy) and the two trees for each cultivar (1 and 2 days after the endo therapy, for Ogliarola salentina and Cima di Melfi, respectively), the metabolites responsible for the intraclass discrimination are essentially the same differentiating the treated plants with respect to day 0 (oleuropein, mannitol, quinic acid) (Supplementary Fig. S6). Interestingly, in the trees comparison, for both cultivars (Ogliarola salentina and Cima di Melfi), significantly higher mannitol and lower oleuropein levels were observed for the samples showing less CoDiRO symptoms (< 25%, Table 3). In the case of the Cima di Melfi cultivar, also a significantly higher level of quinic acid was observed for the samples showing more CoDiRO symptoms (25–50%, Table 3).

**Figure 3:**
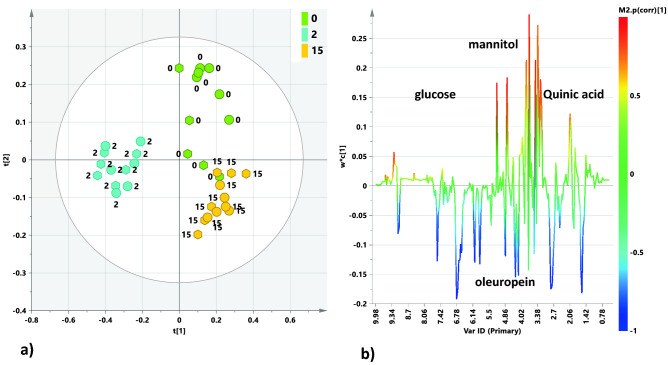
(**a**) PLS-DA t[1][2] scores plot (three components give R2X = 0.574, R2Y = 0.886; Q2 = 0.73) for leaf sample extracts from Ogliarola salentina (circles) and Cima di Melfi (hexagons) tree cultivars at day 0 (before injection), 2, and 15 after the injection. (**b**) Loading line plot for the model coloured according to the correlation-scaled loading (p(corr) ≥|0.5|).

Subsequently, a further supervised PLS-DA analysis was performed. The specific aim was to develop a model able to describe and give useful information, on the discriminating metabolites, for the classes of observations showing the highest and lowest differences with respect to time 0 (before treatment conditions). A PLS-DA model was therefore built by considering, for all the available leaf extracts, besides the time 0 samples, specifically the two time points (2 days and 15 days after treatment), characterized by the highest and lowest discrimination, with respect to pretreatment, respectively (Fig. 3a). The PLS-DA analysis gave a four component models with R2X = 0.574, R2Y = 0.886; Q2 = 0.73. The resulting scores plot demonstrated that day 2 (after treatment) samples class was clearly separated from both day 0 and day 15 along with the first component. Moreover, a certain degree of discrimination between these latter samples classes (day 0 and day 15) was also detected along with the t2 component (Fig. 3a). The specific observed difference between samples from day 0 and day 15 was also confirmed in the pairwise OPLS–DA analysis (Supplementary Fig. S3f). Quinic acid contributes to this differentiation, with higher relative content at day 0 with respect to day 15 (Supplementary Fig. S3g). Again, essentially the same trend was observed considering separately the Ogliarola salentina and Cima di Melfi cultivars (Supplementary Figs. S7 and S8). In order to identify the metabolites contributing to the separation along with the first component, the loading line plot for the PLS-DA model (Fig. 3b) was analyzed. The higher relative content of oleuropein (binned signals at 7.54, 6.78, 6.06, 5.86, 4.26, 2.82, and 1.58 ppm) and its aldehydic forms were observed for day 2 samples. On the contrary, signals related to glucose (5.18 and 4.58 ppm) and mannitol (binned at 3.82, 3.7 ppm) together with quinic acid (2.02 ppm) showed a higher relative content in the pre-treatment samples (day 0). These results agree with our previous findings and literature data reporting an increase of mannitol and quinic acid as a consequence of the infection^2,11,16^. The quantitative estimate of the discriminating power for the Fig. 3b variables was described by the corresponding weight (wc^*^) and the correlation parameter (pcorr) values. Oleuropein, mannitol, quinic acid, and glucose exhibited a strong discriminating contribution to the model with high statistical reliability |pcorr|≥ 0.5. These metabolites could be therefore considered as possible biomarkers in the metabolic profiles of infected trees, discriminating their conditions before and after the endo-therapy. More quantitative estimates of the discriminatory power for each of the variables identified in Fig. 3b were described by the VIP values parameter Table 2.

**Table 2:** List of discriminating chemical descriptors (variables) with corresponding weights (wc*), correlation coefficient (pcorr), and variable importance on the projection (VIP). (terms with VIP ≥ 1 have strong discrimination power).

Metabolite	Var ID (bucket)	M13.w*c[1]	M13.p(corr)[1]	M13.VIP[1]
Oleuropein	1.58	− 0.179971	− 0.959267	2.74124
Quinic acid	2.06	0.118481	0.77162	1.80465
Oleuropein	2.82	− 0.173685	− 0.964654	2.64549
Glucose	3.38	0.280211	0.753741	4.26804
Mannitol	3.82	0.219113	0.763032	3.33743
Mannitol	3.7	0.298225	0.953063	4.54243
Oleuropein	4.26	− 0.150665	− 0.898753	2.29486
Glucose	4.58	0.190089	0.958284	2.89534
Glucose	5.18	0.181498	0.950889	2.7645
Oleuropein	5.86	− 0.129298	− 0.931106	1.96941
Oleuropein	6.06	− 0.127308	− 0.94796	1.93909
Oleuropein	6.78	− 0.189395	− 0.9191	2.88478
Oleuropein	7.54	− 0.125167	− 0.935465	1.90648
Aldehydic form of oleuropein	9.1	− 0.0793029	− 0.750975	1.20791

### Time course analysis of discriminating metabolites

**Figure 4:**
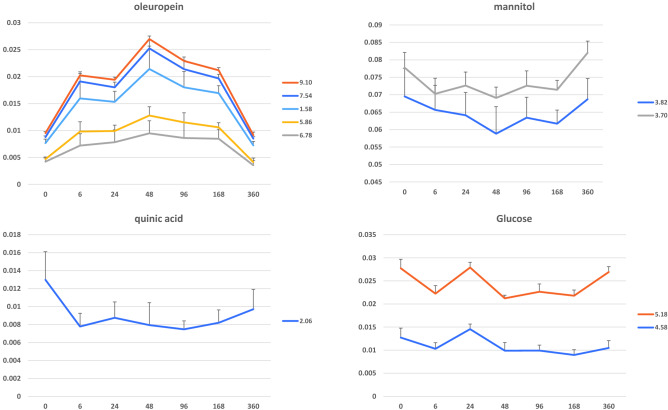
Time course of discriminating metabolites related bucket at different intervals of 0, 0.25, 1, 2, 4, 7, and 15 days after DENTAMET injection. Mean and relative standard error refer to the average bucket intensities for each of the discriminating metabolites.

The time course trend of the metabolic changes occurring after the endo-therapy was also analyzed for the assigned discriminating metabolites. The average bucket intensities for each of the discriminating metabolites were reported as a function of time in Fig. 4. The time course graph for oleuropein related buckets showed the strongest increase occurring 48 h after the injection (day 2), followed by a slow decrease to day 7 and a further strong decline to almost reach, finally (day 15), the pre-treatment levels. An opposite trend was observed for the mannitol related buckets. In this case, a clear decrease was observed from time 0 (before the injection) to the lowest level (day 2). This was followed by a slow increase to day 7 therefore further enhanced to almost reach, finally (day 15), the pre-treatment levels. The trend observed for the selected quinic acid related bucket clearly showed a drastic decrease occurring just 6 h after the injection (day 0.25) and essentially remaining steady over the next 15 days. Some variation could be also observed for glucose levels over the investigated period with an initial minimal decrease within 6 h from the treatment followed by a small peak (at day 1) and a final increase almost to the original levels (from day 7 to day 15).

**Figure 5:**
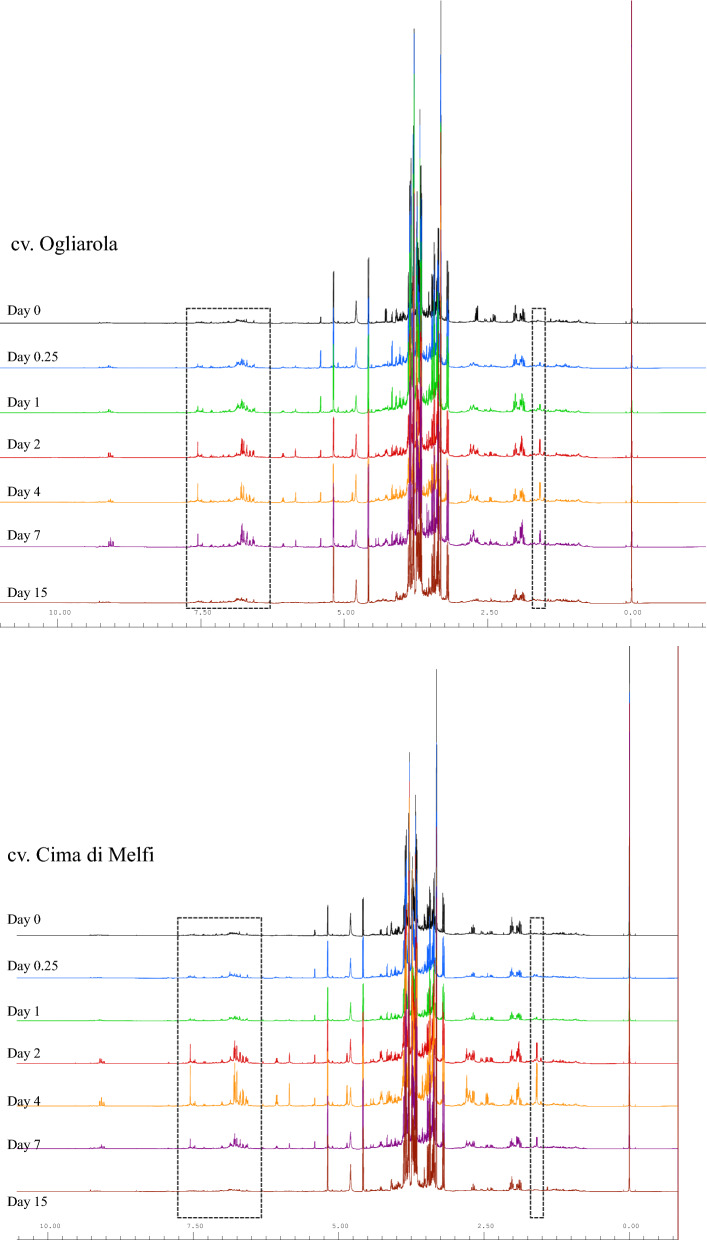
Stacked plot of ^1^H NMR spectra of leaf sample extract at different day from the injection for Ogliarola salentina and Cima di Melfi cultivar trees. The increase of the oleuropein signals intensity was indicated.

The observed general time course trend analysis could be also observed by direct comparison of representative Fig. 5 (or cumulative Supplementary Figs. S9 and S10) leaf samples spectra acquired at different days from the injection for each of the two considered cultivars.

### Time course analysis of Cu and Zn leaf concentrations

**Figure 6:**
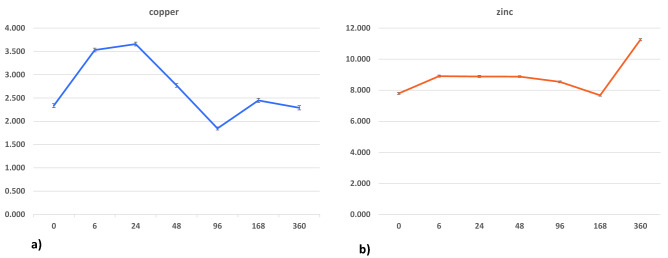
Time course of copper (**a**) and zinc (**b**) leaf concentrations at different intervals (expressed as hours in the graph) of 0, 0.25, 1, 2, 4, 7, and 15 days after DENTAMET injection. The mean and relative standard error refer to the two elements' average concentrations, and the values are expressed as ppm (mg/kg of fresh weight).

The time course trend of the leaf concentration changes occurring after the endo-therapy was also analyzed for Cu and Zn. The average concentrations of the two elements were reported as a function of time in Fig. 6a,b and in Supplementary Table S2. The time course graph of Cu concentration showed the highest value 24 h after the injection (day 1), and then a rapid decrease to day 2 and 4 (with values even lower than those relating to time 0) and a substantial return to concentration values similar to the initial ones, the pre-treatment levels, after 7 and 15 days. A different trend was observed for Zn concentration. In this case, we observed only a slight increase after 6 h (day 0.25). Concentration values remained essentially steady after 1, 2, and 4 days, with a decrease after 7 days with values similar to the pre-treatment levels. A drastic increase was finally observed after 15 days of injection.

## Discussion

In this study, we evaluated a novel proprietary precision injection system (referred to herein as “the TIPS Injection System” available from Invaio Sciences) for controlling OQDS in olive trees by injecting agro-actives into the tree vascular system. The direct effect of DENTAMET trunk injection on the metabolic profiles of *X. fastidiosa* infected trees was analyzed through nuclear magnetic resonance (NMR) for the first time in this study. The experimental design focused on the short-term effect monitoring of endo-therapy by following the time course of metabolic profile changes from time 0 (before the injection) to 15 days after the trunk injection of two local cultivars, Ogliarola salentina and Cima di Melfi. Intermediate samples events were also analyzed at 0.25, 1, 2, 4, and 7 days after the biocomplex application. This study allowed to precisely follow some metabolic changes in the treated trees during a short-time period. Previous studies, indeed, have investigated such changes after foliar applications performed some months before the samplings^2,10,11^. In the present study, all samplings were taken during a defined time-course, thus allowing to obtain a clear profile for the endo therapy injection and the metabolites variation.

Currently, there are no studies on the endo-therapeutic treatment effects on the metabolic profile of plants exposed to various types of stress, including pathogenic infections. To the best of our knowledge, this work represents the first insight into the detection of the immediate effects (metabolic profile changes) for an endo-therapeutic treatment of *X. fastidiosa* infected trees. Metabolomic analysis by NMR spectroscopy showed that the trunk injection of the biocomplex DENTAMET induced relevant modifications in the leaves extracts metabolic profile. These includes a significant temporary increase in oleuropein, probably ascribable to stimulation of the phenylpropanoid pathway. It is known that oleuropein is the main phenolic compound in the leaf methanolic extract and it is well recognized for its human health related properties^15^. Likewise, the increase of oleuropein could be also related to its role in plant protection and resistance against herbivores and pathogens^17^. Phenylpropanoid metabolism is often stimulated when plants are exposed to several environmental constraints^18^.

A recent study investigated the effect of boron (B) foliar application on the phenols concentration, especially oleuropein^19^. The authors observed an increase of these compounds in the leaves of treated olive trees after the boron application. These findings open a new perspective in biochemical farming practices. Specific applications could be used to increase the amount of phenols in olive leaves, enhancing olive pathogen/insect resistance^19^. In this context, the increase in oleuropein content, observed in our study, could be related to the very short-term release of defense molecules, naturally expressed by the plant, in the specific considered season. This is a peculiar consequence of endo-therapy treatment. On the other hand, according to our previous results on foliar treatments, these plant release of particular defense molecules appears to be strongly dependent on the considered season and set timespan comparison^2,10,11,20^. It should be also considered that the pool of phenolic compounds could be characterized by a very complex control mechanism that leads to different responses to treatment application. As described, it is complex to explain the difference observed in oleuropein content after foliar treatment. Nevertheless, it is well known that oleuropein concentration is influenced by abiotic stresses such as cold and salt conditions, and its content in plants increases under these circumstances^17^.

Moreover, important pieces of evidence recently described changes in the amount of some secondary metabolites (phenylpropanoid pathway correlated) and their roles in resistance mechanism against *X. fastidiosa*^21^. Another important result is correlated to the observed clear decrease in the mannitol content as a specific consequence of the endo-therapy. Interestingly this observation is in accord with the recently described higher mannitol amount in *X. fastidiosa*-infected plants with respect to the non-infected ones, suggesting its production as a consequence of the pathogen infection^22^. As known, mannitol is one of the most common sugars in nature, present in most of the vascular plants such as Oleaceae with a specific role as a carbon and energy source for plant growth, as well as an osmoprotectant against biotic and abiotic stress^1^. Thus, the accumulation of mannitol in leaves in response to infection may confer more resistance and tolerance by facilitating osmotic regulation and supporting redox control against ROS induced by *X. fastidiosa*^22,23^.

Moreover, mannitol is also a powerful osmoregulator in leaf mesophyll, relieving *O. europaea* under drought and salinity stress^1^. Interestingly, although, the osmoprotectants accumulation mechanism is not very clear yet, it can be directly or indirectly involved in the tolerance development of plants^1^. Accordingly, the already described increase of mannitol content in response to foliar fertilization^20^ or biocomplex application treatments^2^ of infected plants could be also be related to improved efficiency for physiological performance and photosynthetic capability^20^. Together with mannitol, higher content of glucose observed in the samples collected before the injection was already suggested as a result of a cascade of defense responses against diseases and wounding involving shifts in carbohydrate metabolism to increase carbon uptake^11,24^. Interestingly, among the observed change, the reduction of quinic acid after the injection provided significant findings since this metabolite was already described as a disease biomarker^2,11,21,25^. In particular, modulation of quinic acid levels in response to the pathogenic attack was reported in *X. fastidiosa* infected trees^25^. However, similar metabolic pathways alteration were observed in other *X. fastidiosa* susceptible plants. In particular, an increased level of quinic acid (besides other metabolites) was observed increased in *X. fastidiosa* infected grapevine plants in comparison to non-infected^26^. Moreover, analysis of Cu and Zn leaf concentration confirmed that DENTAMET endo-therapy injection releases such ions within olive foliage, thus confirming the data already observed through its foliar application^9^. However, we also observed a different trend shown by the ions. After an initial increase, Cu concentration starts to decrease from the second to the fourth day after the injection. By contrast, after an initial steady phase, the Zn concentration suddenly increases 15 days after the injection. It is difficult to explain such quite opposite trend that deserves further investigation. It has been observed, however, that Zn actively participates in the synthesis of the hormone auxin that incites plant growth^27^, so that an accumulation in the olive leaves could be putatively hypothesized upon its supply through the injection. Nevertheless, transcriptomic sequencing^28^ or gene expression analyses could also provide some further clues to explain the metabolic data interpretation, including metal ions levels, in bacterium-affected trees as well as in treatment response.

This study has also found that each single olive tree of both cultivars, Ogliarola salentina, and Cima di Melfi, showed a different metabolic pattern upon the injection, thus representing an additional issue to consider for the management of *X. fastidiosa* subsp. *pauca* through endo-therapy*.* The influence of some abiotic parameters such as habitat and rock type on single tree growth response to fertilization was previously observed in forest plant species^29,30^. This finding also deserves future studies to precisely calibrate the right dose of active ingredient to supply to every single tree, according to some other parameters (i.e., tree age, soil type, fertilization scheme) other than the occurrence of *X. fastidiosa* subsp. *pauca* symptoms.

It should be finally considered that being more precisely applied, and need lower doses of an active ingredient, endo-therapy results in a desirable choice to treat the olive quick decline syndrome. Moreover, the development of a precise, high throughput injection system, that can deliver the active ingredients into the plant’s vessels resulted crucial for understanding in real time biomarker changes upon administration of agro-actives.

## Conclusions

In this work we performed, for the first time, short-term monitoring of metabolic pathways reprogramming for infected olive trees after precision intravascular delivery of a Cu/Zn citric acid biocomplex (DENTAMET) using the TIPS Injection System. The ^1^H NMR metabolomics approach showed, after the injection, a significant decrease of the disease biomarker quinic acid with simultaneous increase of polyphenols and oleuropein related compounds, in the leaves extracts. Among the observed changes, for infected plants with respect to the before injection conditions, a decrease of the reducing sugars mannitol and glucose was also observed. Interestingly, increased levels of both sugars were already reported because of infection. Nevertheless, the seasonality effect, as well as the sampling period with the specific period considered for comparison, should be strongly taken into account when analyzing these results with respect to literature data.

Although the herein reported observations are related to preliminary experimental treatment, the NMR-based methodology here used for the first time appears to be pivotal for short-term monitoring after endo therapy. The obtained results on the metabolic reprogramming of the trees upon the treatment were further supported by analytical information on the zinc and copper level in the leaves. These latter clearly indicated that both ions have reached the tree leaves buttressing the correlation of the observed metabolic changes with the performed therapeutic treatment. Certainly, further studies should be undertaken to clearly identify all the factors influencing the observed changes and the specific metabolic pathways underlining the reported effects. Further work is in progress to optimize treatment doses and frequencies and to obtain long-term results able to confirm possible developments towards pathologic remission.

## Methods

### Endo-therapy device

**Figure 7:**
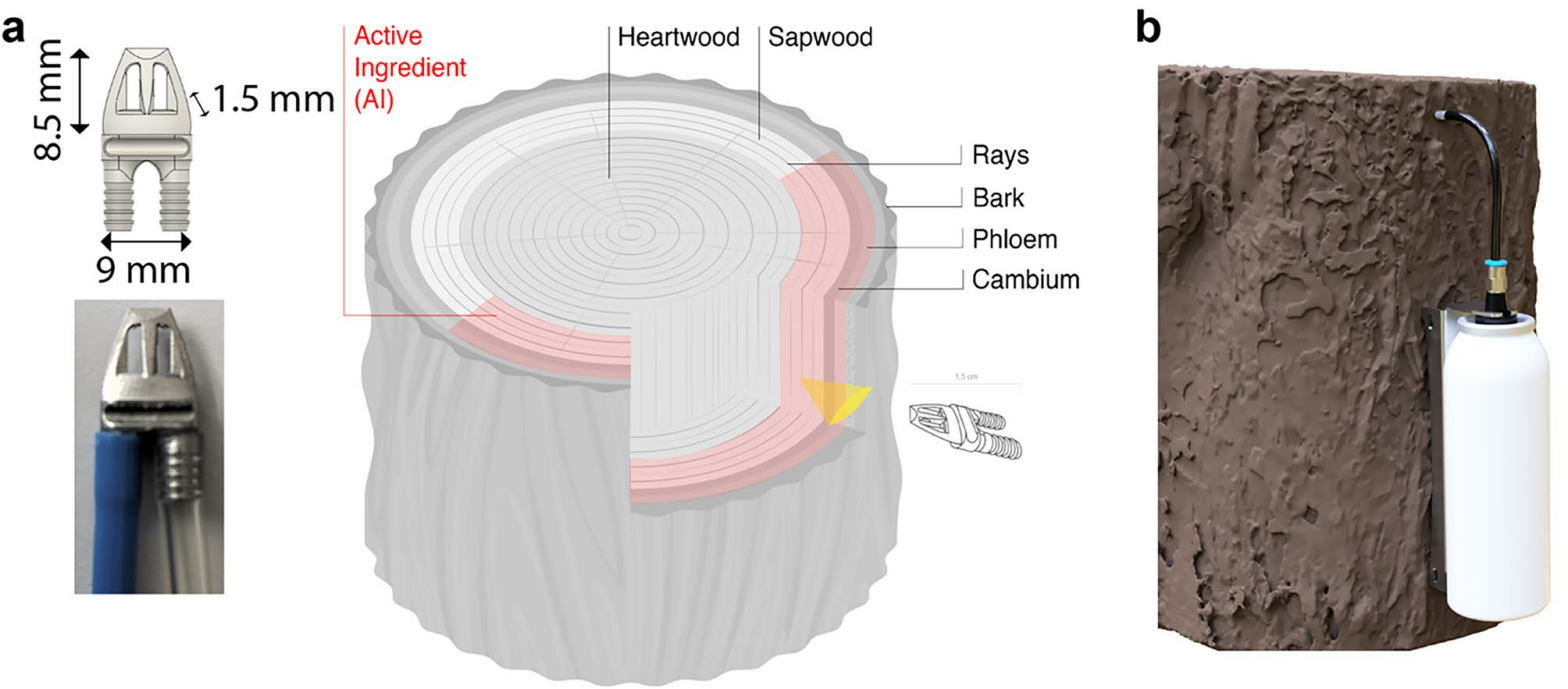
Endotherapy device (**a**) An arrow-shaped metal component of the TIPS Injection System was designed to inject active ingredients precisely in the plant vascular system of olive trees. (**b**) Modular spray-can device of the TIPS Injection System applied to an olive tree. The system allows a low-pressure release of compounds.

The device used for the endo-therapy application is depicted in Fig. 7. It contains an extremely thin and arrow-shaped metal tip designed for precise delivery of the active ingredients into the plant vascular system (Fig. 7a). The reduced tip dimensions (8.5 mm length, 9 mm wide, and 1.5 mm thick, are planned for minimal damage to the plants. Thanks to its precise design with cavities against the insert direction, clogging of the system is normally prevented. The tip is very robust, durable, leakage free, and designed for multiple applications. The device also encloses a pre-pressurized canister with a bag-on-valve (BOV) containing the agro-active, which is usually released into the vascular system within hours (Fig. 7b). For tree injection, after removing the bark, the tip is inserted into the vascular system with the use of a tip adapter and a hammer until it is properly sealed. The can holder is then attached to the tree and connected to the tip tubing. By a simple click-in, the pre-loaded canister is inserted into the can holder, effectively releasing the liquid into the plant vessels. The overall execution takes place within seconds (~ 30–60 s).

### DENTAMET microinjection

DENTAMET is a fertilizer containing zinc (4% w/w) and copper (2% w/w) salt complexed with hydracid of citric acid obtained through a fermentation process^10^. Four olive trees, coded 704.1, 704.2, 704.3, and 704.4, grown as a crop and located in a CoDiRO-exhibiting olive tree orchard (40° 40′ 35.0″ N; 17° 38′ 47.2″ E), in the district of Carovigno (Brindisi, Apulia province, South East Italy), were injected with 50 ml of a water solution containing 20% DENTAMET (v/v), on September 3rd, 2020, on the Southside of the tree. The average values of the main climatic factors parameters, measured at the nearest wheatear station^31^ (40° 44′ 35.0″ N; 17° 25′ 33″ E), are reported in Supplementary Table S3. The above-described TIPS Injection System was used for this specific treatment. Approximately 2 cm of bark was removed using a multitool kit, and the microinjection tip was introduced into the trunk of the tree. A spring-loaded syringe containing the specific volume of the treatment solution was then connected to the tip, and the product was released into the tree at low pressure (1.4–2.7 bars). The age, cultivar, location, disease conditions, and dose of injected DENTAMET are reported, for all the studied trees, in Table 3.

**Table 3:** List of the experimental trials conditions for the studied trees. Symptoms incidence was visually assessed^9^ and PCR analyses^7^.

Tree number	Cultivar	Location	Volume (ml) Total (DENTAMET)	Age (years)	Symptoms of olive quick decline syndrome
704.1	Cima di Melfi	Carovigno	50 (10)	30	< 25%
704.2	Cima di Melfi	Carovigno	50 (10)	30	25–50%
704.3	Ogliarola salentina	Carovigno	50 (10)	80	< 25%
704.4	Ogliarola salentina	Carovigno	50 (10)	80	25–50%

### Leaves sampling

Three samples constituted by a statistically representative number of mature leaves (20 leaves) were collected on going from the North to the Southside of the full canopy for each of the four trees at day 0 (before injection) and 0.25, 1, 2, 4, 7 and 15 days after the injection. All the 84 total collected leaf samples were placed in coded plastic bags into a refrigerated box and taken to the laboratory. Samples were therefore stored at − 20 °C until metabolomic analysis. Associated symptoms incidence for the examined trees were visually assessed and further confirmed by PCR analyses according to Harper et al.^32^. PCR analyses showed that all trees, sampled according to Tatulli et al.^7^, resulted infected by *X*. *fastidiosa* subsp. *pauca*. We declare that we have the permission to collect leaves samples according to a specific agreement between Invaio Sciences (D.V.) and the orchard owner. The samples were collected in accordance with relevant institutional, national, and international guidelines and legislation. The formal identification of the plant material was assessed by Marco Scortichini (M.S.) from the olive orchard structure records supplied by the farmer and confirmed by pomological characterization^33^. Voucher samples of the lyophilized studied plant material are available from the authors (F.P.F.).

### Sample preparation for ^1^H NMR analysis

Samples were prepared according to the experimental procedure as reported in the literature^34^. Briefly, olive leaf samples (each one containing 20 leaves) were plunged into liquid N_2_ and ground to a fine powder with a stainless-steel blender. Ground leaves were transferred into a plastic tube and placed in a freeze dryer for 48 h. Lyophilized plant material (100 mg) was weighted into an autoclaved 2 ml Eppendorf tube. Thereafter, 0.75 ml of CD_3_OD and 0.75 ml of KH_2_PO_4_ buffer in D_2_O (pH 5.9) containing 0.05% w/v TSP-d4 (sodium salt of trimethylsilylpropionic acid) were added to each sample. The content of the Eppendorf tubes was mixed thoroughly with a vortex mixer at room temperature for 1 min and then sonicated for 10 min at room temperature. The tubes were therefore spun in a microcentrifuge at 17,000*g* for 20 min. Then, for each sample, 600 µl of the supernatant was filled into a 5 mm NMR tube.

### ^1^H-NMR spectra acquisition and processing

All NMR spectra acquisition were performed at 300 K on a Bruker Avance III 600 MHz Ascend NMR Spectrometer (Bruker Italia, Milano, Italy), operating at 600.13 MHz, equipped with a TCI cryoprobe (inverse Triple Resonance Cryoprobe Prodigy), incorporating a z-axis gradient coil and automatic tuning-matching (ATM). Experiments were run in automation mode after loading samples on an integrated Bruker Automatic Sample Changer, interfaced with IconNMR software (Bruker). For each sample, a ^1^H NMR spectrum was acquired using water signal suppression (Bruker pulseprogram zgcppr), the spectral window of 20.0276 ppm (12,019.230 Hz), 90° pulse of 7.620 µs, and 64 scans. The standard FID processing procedures, such as the Fourier transform, phase and baseline correction, and 0.3 Hz line broadening, were carried out using TopSpin 3.5 (Bruker, Biospin, Italy). All the NMR spectra were calibrated to the internal standard TSP (δ = 0.00 ppm). The characterization of the metabolites was also supported by two-dimensional homo- and heteronuclear NMR spectra (2D ^1^H J-resolved, ^1^H COSY, ^1^H–^13^C HSQC, and HMBC) and comparison with the literature data^2,10,11,16,35^.

### Multivariate statistical analysis

For chemometric studies, NMR spectra were segmented, into histograms, with a fixed base width of 0.04 ppm ("normal rectangular bucketing") by Amix 3.9.15 (Analysis of Mixture, Bruker BioSpin GmbH, Rheinstetten, Germany) software. The obtained buckets were subjected to mean centering and Pareto scaling methods. The total sum normalization was applied to minimize small differences due to metabolites concentration and/or experimental conditions among samples^36–38^. All aligned buckets row reduced spectra, labeled with the value of the central chemical shift for its specific 0.04 ppm width, were used for multivariate data analysis with the help of the SIMCA-P version 14 (Sartorius Stedim Biotech, Umeå, Sweden) software. The signals’ related buckets areas are reported in Supplementary Table S4. The Principal Components Analysis (PCA) and the Partial Least-Squares Discriminant Analysis (PLS-DA) or Orthogonal Projections to Latent Structures Discriminant Analysis (OPLS-DA) were performed as unsupervised and supervised methods respectively. PCA is aimed at extracting the maximum possible information from a multivariate data structure, summarizing it in a few linear combinations of the variables themselves^39^. The PCA is used, to obtain a general description of the sample distribution and their possible grouping in clusters^39^. The assessment of the correlation between the distribution of the clusters of the analyzed samples and the considered classes is therefore carried out by using supervised Partial Least-Squares Discriminant Analysis (PLS-DA) or Orthogonal Projections to Latent Structures Discriminant Analysis, (OPLS-DA). The PLS-DA is the regression extension of PCA, which gives the maximum covariance between the measured data (X variable, matrix of buckets related to metabolites in NMR spectra) and the response variable (Y variable, matrix of data related to the class membership). The OPLS-DA is a modification of the PLS-DA method which filters out variation not directly related to the focused discriminating response, by separating the portion of the variance useful for predictive purposes from the non-predictive variance^40,41^. The validity and the degree of overfitting for OPLS models were checked by using the internal cross-validation default method (sevenfold) and with permutation test^38^. The quality of the models was assessed by R2, Q2 parameters. The first (R2) is a cross-validation parameter defined as the explained variance of the models and indicates the goodness of fit. The second (Q2) describes the portion of the variance in the data predictable by the model^42^. The variables responsible for the observed discrimination were identified by using the statistical tool S-line plot. The S-line plot is tailor-made for NMR spectroscopy data and creates a plot of the loading vectors discriminating the classes along with the considered component. Loading vectors are usually colored according to their absolute correlation scaled value, p(corr)[1]^36^.

### Metal characterization

Element concentrations in olive leaf samples were measured using the Inductively Coupled Plasma Atomic Emission Spectroscopy (ICP-AES). The spectrometer was an ICAP 6300 with a Dual view, empowered by iTEVA software (Thermo Scientific, Waltham, MA, USA). Each leaf sample was analyzed by following the standard procedures^9,43^. Briefly, 1 g of olive leaves was mixed with 4 ml of H_2_O_2_ and 6 ml of super pure HNO_3_ at 180 °C for 10 min, using a microwave digestion system (Milestone Start D). Then it was cooled, diluted with super pure water to a final volume of 20 ml, filtered through Whatman No. 42 filter papers, and measured for elemental content using an ICP spectrometer. The spectrometer was previously calibrated for quantitative analysis with five standard solutions containing known concentrations (0.001, 0.01, 0.1, 0.5, and 1.0 mg/l) of the elements. The calibration lines showed correlation coefficients (r) greater than 0.99 for all the measured elements. The results were expressed as the average of three different measurements, and the element concentrations were expressed as ppm (mg/kg of sample weight).

## Supplementary Information

Supplementary Information.

## Data Availability

All data generated or analyzed during this study are included in this published article (and its Supplementary Information files).
