# Supplementary material for: Agro-active endo-therapy treated Xylella fastidiosa subsp. pauca-infected olive trees assessed by the first 1H-NMR-based metabolomic study
Source: Sci Rep. 2022 Apr 8;12:5973. doi: 10.1038/s41598-022-09687-8 (PMC8993878; doi:10.1038/s41598-022-09687-8)
Supplement: Supplementary file 1 — Supplementary Information. [file 41598_2022_9687_MOESM1_ESM.docx]

**Supplementary Information for:**

**Agro-active endo-therapy treated *Xylella fastidiosa* subsp. *pauca*- infected olive trees assessed by the first ^1^H-NMR based metabolomic study**

**Chiara Roberta Girelli^1^, Mudassar Hussain^1^,** **Dimitri Verweire^2^, Michael Oehl^2^, Josep Massana-Codina^2^, Maier S. Avendaño^2^, Danilo Migoni^1^, Marco Scortichini^3^ and Francesco Paolo Fanizzi^1,*^**

^1^Department of Biologic and Environmental Sciences and Technologies, University of Salento, 73100 Lecce, Italy

^2^Invaio Sciences, Cambridge, MA 02138, USA

^3^Research Centre for Olive, Fruit and Citrus Crops, Council for Agricultural Research and

Economics (CREA), 00134 Roma, Italy.

^*^fp.fanizzi@unisalento.it

**Figure S1**: PCA t[1][2] scores plot (three components give R2X = 0.768 Q2 = 0.69) for leaf samples from Ogliarola salentina (circles) tree cultivars tree at day 0 (before injection) and 0.25, 1, 2, 4, 7 and 15 after injection. Sample symbols are colored according to different days from the treatment.

**Figure S2**: PCA t[1][2] scores plot (three components give R2X = 0.72, Q2 = 0.59) for leaf samples from Cima di Melfi (hexagons) tree cultivars tree at day 0 (before injection) and 0.25, 1, 2, 4, 7 and 15 after injection. Sample symbols are colored according to different days from the treatment.

**Figure S3:** Pair wise OPLS-DA scores plots or leaf samples from Ogliarola (circles) and Cima di Melfi (hexagons) tree cultivars comparing time 0 (before injection) with 0.25 (a), 1 (b), 2 (c), 4 (d), 7 (e) and 15 (f) days after the injection. g) S line plot for the 0 *vs* 15 model

**Figure S4:** Pair wise OPLS-DA scores plots or leaf samples from Ogliarola (circles) tree cultivars comparing time 0 (before injection) with 0.25 **a**), 1 **b**), 2 **c**), 4 **d**), 7 **e**) and 15 **f**) days after the injection.

**Figure S5:** Pair wise OPLS-DA scores plots for leaf samples from Cima di Melfi (hexagons) tree cultivars comparing time 0 (before injection) with 0.25 **a**), 1 **b**), 2 **c**), 4 **d**), 7 **e**) and 15 **f**) days after the injection.

**Figure S6:** Pairwise OPLS- DA and related S line plots comparing the two cultivars **a)** and **b**) and the two trees for Ogliarola salentina (circles) **c)** and **d**) and Cima di Melfi (hexagons) **e)** and **f**) of treated plants (after 1 and 2 days after the endo therapy).

**Figure S7: a)** PLS - DA t[1][2] scores plot (three components give R2X= 0.763, R2Y=0.945; Q2= 0.856) for leaf sample extracts from Ogliarola (circles) tree cultivars at day 0 (before injection), 2, and 15 after the injection. **b**) Loading line plot for the model coloured according to the correlation-scaled loading (p(corr) ≥|0.5|).

**Figure S8: a)** PLS - DA t[1][2] scores plot (three components give R2X= 0.574, R2Y=0.886; Q2= 0.73) for leaf sample extracts from Cima di Melfi (hexagons) tree cultivars at day 0 (before injection), 2, and 15 after the injection. **b**) Loading line plot for the model coloured according to the correlation-scaled loading (p(corr) ≥|0.5|).

**Figure S9:** Stacked plot of ^1^H NMR spectra of leaf sample extract at different day from the injection for Ogliarola salentina cultivar trees.

**Figure S10:** Stacked plot of ^1^H NMR spectra of leaf sample extract at different day from the injection for Cima di Melfi cultivar trees.

**Table S1.** Statistical parameters of supervised OPLS-DA models comparing time 0 with each of the considered times. R2X and R2Y indicate the fraction of variance of the X and Y matrix, respectively. Q2 is a goodness of prediction parameter representing the portion of variance in the data predictable by the model.

| **OPLS-DA (1+1+0)** | **R2X** | **R2Y** | **Q2** |
| --- | --- | --- | --- |
| **Ogliarola salentina** |  |  |  |
| Days 0 *vs* 0.25 | 0.74 | 0.904 | 0.871 |
| Days 0 *vs* 1 | 0.803 | 0.892 | 0.832 |
| Days 0 *vs* 2 | 0.801 | 0.982 | 0.967 |
| Days 0 *vs* 4 | 0.631 | 0.946 | 0.898 |
| Days 0 *vs* 7 | 0.772 | 0.985 | 0.869 |
| Days 0 *vs* 15 | 0.508 | 0.804 | 0.694 |
| **Cima di Melfi** |  |  |  |
| Days 0 *vs* 0.25 | 0.631 | 0.908 | 0.808 |
| Days 0 *vs* 1 | 0.561 | 0.921 | 0.814 |
| Days 0 *vs* 2 | 0.643 | 0.954 | 0.904 |
| Days 0 *vs* 4 | 0.684 | 0.985 | 0.857 |
| Days 0 *vs* 7 | 0.558 | 0.888 | 0.782 |
| Days 0 *vs* 15 | 0.523 | 0.99 | 0.726 |

**Table S2.** Copper (Cu) and Zinc (Zn) leaf concentration values, measured at different intervals of 0, 0.25, 1, 2, 4, 7 and 15 days after Dentamet injection. Mean and relative standard error refers to the average concentrations of the two elements and the values are expressed as ppm (mg/kg of fresh weight).

| **Time (h)** | **[Cu] (ppm)** | **± S.D.** | **[Zn] (ppm)** | **± S.D.** |
| --- | --- | --- | --- | --- |
| **0** | 2.335 | 0.043 | 7.795 | 0.061 |
| **6** | 3.529 | 0.040 | 8.908 | 0.070 |
| **24** | 3.656 | 0.041 | 8.884 | 0.067 |
| **48** | 2.770 | 0.039 | 8.878 | 0.060 |
| **96** | 1.845 | 0.033 | 8.539 | 0.061 |
| **168** | 2.446 | 0.043 | 7.670 | 0.054 |
| **360** | 2.290 | 0.046 | 11.254 | 0.068 |

**Table S3.** Average of monthly cumulative rainfall (mm), temperature (°C), relative humidity (%),wind speed (m/s) and solar radiation(W/m^2^) are reported and calculated for each month in 2020.

|  | **temperature** | **relative**  **humidity** | **rain** | **wind** | **solar**  **radiation** |
| --- | --- | --- | --- | --- | --- |
| **January** | °C | % | mm | m/s | W/m2 |
| **February** | 8.98 | 70.87 | 0.07 | NaN | 128.84 |
| **March** | 9.40 | 77.70 | 0.12 | NaN | 162.86 |
| **April** | 12.66 | 64.18 | 0.10 | NaN | 220.22 |
| **May** | 17.30 | 63.03 | 0.03 | 3.07 | 251.13 |
| **June** | 20.62 | 65.19 | 0.07 | NaN | 278.70 |
| **July** | 23.86 | 55.75 | 0.00 | 2.70 | 314.62 |
| **August** | 25.54 | 59.84 | 0.00 | 2.49 | 276.31 |
| **September** | 22.22 | 73.04 | 0.14 | 2.83 | 196.17 |
| **October** | 13.92 | 82.91 | 0.07 | 2.54 | 135.18 |
| **November** | 12.06 | NaN | 0.22 | 2.22 | 80.97 |
| **December** | 8.90 | 90.98 | 0.10 | 2.39 | 63.12 |

**Table S4.** Assignment of relevant metabolites in the ^1^H NMR zgcppr spectrum of olive leaf extracts. Signals’ related buckets areas, integrated at different times are indicated. OLE: oleuropein; SUC: sucrose; GLU: glucose; MAN: mannitol; QUI: quinic acid

|  | ppm | 9.1 | 7.54 | 6.78 | 6.06 | 5.86 | 5.42 | 5.18 | 4.58 | 4.26 | 3.82 | 3.7 | 3.38 | 2.82 | 2.02 | 1.58 |
| --- | --- | --- | --- | --- | --- | --- | --- | --- | --- | --- | --- | --- | --- | --- | --- | --- |
| **day** | **tree** | **OLE** | **OLE** | **OLE** | **OLE** | **OLE** | **SUC** | **GLU** | **GLU** | **OLE** | **MAN** | **MAN** | **GLU** | **OLE** | **QUI** | **OLE** |
| **0** | **704.1** | 0.00065 | 0.00130 | 0.00423 | 0.00021 | 0.00044 | 0.00116 | 0.01791 | 0.01563 | 0.00374 | 0.06914 | 0.08475 | 0.06456 | 0.00226 | 0.01033 | 0.00284 |
| **0** | **704.1** | 0.00048 | 0.00147 | 0.00362 | 0.00057 | 0.00079 | 0.00207 | 0.01510 | 0.01251 | 0.00274 | 0.06584 | 0.07855 | 0.05204 | 0.00266 | 0.00724 | 0.00394 |
| **0** | **704.1** | 0.00057 | 0.00138 | 0.00392 | 0.00039 | 0.00062 | 0.00161 | 0.01650 | 0.01407 | 0.00324 | 0.06749 | 0.08165 | 0.05830 | 0.00246 | 0.00878 | 0.00339 |
| **0** | **704.2** | 0.00081 | 0.00110 | 0.00453 | 0.00020 | 0.00033 | 0.00205 | 0.01121 | 0.00886 | 0.00263 | 0.05918 | 0.06933 | 0.04552 | 0.00399 | 0.01721 | 0.00370 |
| **0** | **704.2** | 0.00090 | 0.00128 | 0.00475 | 0.00052 | 0.00073 | 0.00249 | 0.01486 | 0.01275 | 0.00410 | 0.06796 | 0.07545 | 0.05416 | 0.00293 | 0.01349 | 0.00290 |
| **0** | **704.2** | 0.00086 | 0.00119 | 0.00464 | 0.00036 | 0.00053 | 0.00227 | 0.01303 | 0.01081 | 0.00337 | 0.06357 | 0.07239 | 0.04984 | 0.00346 | 0.01535 | 0.00330 |
| **0** | **704.3** | 0.00108 | 0.00084 | 0.00448 | 0.00024 | 0.00036 | 0.00367 | 0.01624 | 0.01370 | 0.00302 | 0.07140 | 0.07757 | 0.05454 | 0.00258 | 0.01593 | 0.00224 |
| **0** | **704.3** | 0.00044 | 0.00073 | 0.00293 | 0.00025 | 0.00043 | 0.00560 | 0.01606 | 0.01451 | 0.00338 | 0.08680 | 0.07928 | 0.05351 | 0.00125 | 0.01429 | 0.00160 |
| **0** | **704.3** | 0.00076 | 0.00079 | 0.00371 | 0.00024 | 0.00039 | 0.00463 | 0.01615 | 0.01411 | 0.00320 | 0.07910 | 0.07842 | 0.05403 | 0.00191 | 0.01511 | 0.00192 |
| **0** | **704.4** | 0.00050 | 0.00113 | 0.00339 | 0.00034 | 0.00051 | 0.00208 | 0.01395 | 0.01182 | 0.00407 | 0.07138 | 0.07910 | 0.05186 | 0.00261 | 0.01256 | 0.00320 |
| **0** | **704.4** | 0.00118 | 0.00105 | 0.00571 | 0.00003 | 0.00010 | 0.00152 | 0.01475 | 0.01203 | 0.00193 | 0.06432 | 0.07823 | 0.05940 | 0.00401 | 0.01283 | 0.00337 |
| **0** | **704.4** | 0.00084 | 0.00109 | 0.00455 | 0.00016 | 0.00031 | 0.00180 | 0.01435 | 0.01193 | 0.00300 | 0.06785 | 0.07867 | 0.05563 | 0.00331 | 0.01269 | 0.00329 |
| **0.25** | **704.1** | 0.00032 | 0.00254 | 0.00477 | 0.00114 | 0.00145 | 0.00326 | 0.01075 | 0.00836 | 0.00360 | 0.06254 | 0.07031 | 0.04571 | 0.00326 | 0.00564 | 0.00490 |
| **0.25** | **704.1** | 0.00052 | 0.00282 | 0.00614 | 0.00154 | 0.00195 | 0.00297 | 0.01134 | 0.00893 | 0.00461 | 0.06231 | 0.07005 | 0.04562 | 0.00402 | 0.00569 | 0.00586 |
| **0.25** | **704.1** | 0.00042 | 0.00268 | 0.00545 | 0.00134 | 0.00170 | 0.00312 | 0.01105 | 0.00865 | 0.00410 | 0.06242 | 0.07018 | 0.04566 | 0.00364 | 0.00566 | 0.00538 |
| **0.25** | **704.2** | 0.00083 | 0.00560 | 0.00900 | 0.00495 | 0.00593 | 0.00400 | 0.01004 | 0.00800 | 0.00843 | 0.06050 | 0.06347 | 0.04043 | 0.00944 | 0.00730 | 0.01071 |
| **0.25** | **704.2** | 0.00066 | 0.00219 | 0.00472 | 0.00118 | 0.00144 | 0.00392 | 0.01373 | 0.01148 | 0.00389 | 0.07250 | 0.07696 | 0.05039 | 0.00302 | 0.00973 | 0.00382 |
| **0.25** | **704.2** | 0.00074 | 0.00389 | 0.00686 | 0.00307 | 0.00369 | 0.00396 | 0.01189 | 0.00974 | 0.00616 | 0.06650 | 0.07021 | 0.04541 | 0.00623 | 0.00851 | 0.00726 |
| **0.25** | **704.3** | 0.00144 | 0.00172 | 0.00639 | 0.00077 | 0.00104 | 0.00473 | 0.01414 | 0.01192 | 0.00424 | 0.07526 | 0.07541 | 0.05284 | 0.00365 | 0.00878 | 0.00323 |
| **0.25** | **704.3** | 0.00139 | 0.00159 | 0.00649 | 0.00082 | 0.00105 | 0.00539 | 0.01383 | 0.01190 | 0.00484 | 0.07414 | 0.07117 | 0.04942 | 0.00333 | 0.00858 | 0.00281 |
| **0.25** | **704.3** | 0.00142 | 0.00165 | 0.00644 | 0.00079 | 0.00105 | 0.00506 | 0.01398 | 0.01191 | 0.00454 | 0.07470 | 0.07329 | 0.05113 | 0.00349 | 0.00868 | 0.00302 |
| **0.25** | **704.4** | 0.00145 | 0.00453 | 0.01015 | 0.00350 | 0.00417 | 0.00334 | 0.01000 | 0.00783 | 0.00710 | 0.05948 | 0.06601 | 0.04309 | 0.00862 | 0.00811 | 0.00977 |
| **0.25** | **704.4** | 0.00236 | 0.00417 | 0.00993 | 0.00327 | 0.00381 | 0.00293 | 0.01172 | 0.00917 | 0.00643 | 0.05858 | 0.06865 | 0.04772 | 0.00753 | 0.00839 | 0.00815 |
| **0.25** | **704.4** | 0.00191 | 0.00435 | 0.01004 | 0.00338 | 0.00399 | 0.00314 | 0.01086 | 0.00850 | 0.00676 | 0.05903 | 0.06733 | 0.04540 | 0.00807 | 0.00825 | 0.00896 |
| **1** | **704.1** | 0.00060 | 0.00341 | 0.00643 | 0.00244 | 0.00294 | 0.00306 | 0.01178 | 0.00977 | 0.00592 | 0.05878 | 0.06635 | 0.04504 | 0.00468 | 0.00583 | 0.00654 |
| **1** | **704.1** | 0.00103 | 0.00232 | 0.00676 | 0.00133 | 0.00161 | 0.00186 | 0.01292 | 0.01052 | 0.00470 | 0.06006 | 0.07170 | 0.04882 | 0.00429 | 0.00669 | 0.00483 |
| **1** | **704.1** | 0.00082 | 0.00286 | 0.00659 | 0.00188 | 0.00227 | 0.00246 | 0.01235 | 0.01015 | 0.00531 | 0.05942 | 0.06903 | 0.04693 | 0.00448 | 0.00626 | 0.00568 |
| **1** | **704.2** | 0.00142 | 0.00186 | 0.00640 | 0.00093 | 0.00121 | 0.00290 | 0.01481 | 0.01247 | 0.00475 | 0.07027 | 0.07709 | 0.05402 | 0.00354 | 0.01073 | 0.00384 |
| **1** | **704.2** | 0.00105 | 0.00196 | 0.00574 | 0.00092 | 0.00116 | 0.00239 | 0.01417 | 0.01173 | 0.00422 | 0.06788 | 0.07665 | 0.05144 | 0.00340 | 0.00876 | 0.00398 |
| **1** | **704.2** | 0.00123 | 0.00191 | 0.00607 | 0.00092 | 0.00119 | 0.00264 | 0.01449 | 0.01210 | 0.00448 | 0.06907 | 0.07687 | 0.05273 | 0.00347 | 0.00974 | 0.00391 |
| **1** | **704.3** | 0.00165 | 0.00210 | 0.00762 | 0.00128 | 0.00156 | 0.00412 | 0.01418 | 0.01192 | 0.00493 | 0.07075 | 0.07494 | 0.05305 | 0.00407 | 0.01028 | 0.00382 |
| **1** | **704.3** | 0.00156 | 0.00190 | 0.00725 | 0.00093 | 0.00115 | 0.00370 | 0.01426 | 0.01199 | 0.00449 | 0.07124 | 0.07463 | 0.05341 | 0.00421 | 0.01020 | 0.00374 |
| **1** | **704.3** | 0.00161 | 0.00200 | 0.00743 | 0.00111 | 0.00135 | 0.00391 | 0.01422 | 0.01195 | 0.00471 | 0.07100 | 0.07479 | 0.05323 | 0.00414 | 0.01024 | 0.00378 |
| **1** | **704.4** | 0.00229 | 0.00424 | 0.01264 | 0.00317 | 0.00375 | 0.00209 | 0.01210 | 0.00952 | 0.00788 | 0.05535 | 0.06836 | 0.04821 | 0.00849 | 0.00934 | 0.00839 |
| **1** | **704.4** | 0.00156 | 0.00382 | 0.00975 | 0.00291 | 0.00336 | 0.00218 | 0.01253 | 0.00991 | 0.00564 | 0.05863 | 0.07128 | 0.04902 | 0.00788 | 0.00812 | 0.00807 |
| **1** | **704.4** | 0.00193 | 0.00403 | 0.01119 | 0.00304 | 0.00355 | 0.00214 | 0.01231 | 0.00972 | 0.00676 | 0.05699 | 0.06982 | 0.04862 | 0.00819 | 0.00873 | 0.00823 |
| **2** | **704.1** | 0.00098 | 0.00451 | 0.00788 | 0.00344 | 0.00396 | 0.00225 | 0.01190 | 0.00905 | 0.00598 | 0.05879 | 0.07122 | 0.04897 | 0.00714 | 0.00654 | 0.00901 |
| **2** | **704.1** | 0.00079 | 0.00264 | 0.00584 | 0.00184 | 0.00182 | 0.00234 | 0.01112 | 0.00853 | 0.00412 | 0.06014 | 0.07034 | 0.04510 | 0.00447 | 0.00680 | 0.00711 |
| **2** | **704.1** | 0.00089 | 0.00357 | 0.00686 | 0.00264 | 0.00289 | 0.00229 | 0.01151 | 0.00879 | 0.00505 | 0.05946 | 0.07078 | 0.04704 | 0.00580 | 0.00667 | 0.00806 |
| **2** | **704.2** | 0.00219 | 0.00404 | 0.01064 | 0.00318 | 0.00369 | 0.00275 | 0.01153 | 0.00935 | 0.00826 | 0.05859 | 0.06757 | 0.04722 | 0.00754 | 0.00891 | 0.00823 |
| **2** | **704.2** | 0.00217 | 0.00335 | 0.00996 | 0.00259 | 0.00272 | 0.00266 | 0.01100 | 0.00892 | 0.00758 | 0.05837 | 0.06621 | 0.04473 | 0.00622 | 0.01184 | 0.00806 |
| **2** | **704.2** | 0.00218 | 0.00369 | 0.01030 | 0.00288 | 0.00320 | 0.00271 | 0.01126 | 0.00914 | 0.00792 | 0.05848 | 0.06689 | 0.04597 | 0.00688 | 0.01037 | 0.00814 |
| **2** | **704.3** | 0.00196 | 0.00294 | 0.00866 | 0.00242 | 0.00280 | 0.00439 | 0.01224 | 0.01006 | 0.00665 | 0.06518 | 0.06739 | 0.04789 | 0.00575 | 0.00789 | 0.00634 |
| **2** | **704.3** | 0.00179 | 0.00221 | 0.00744 | 0.00167 | 0.00164 | 0.00386 | 0.01251 | 0.00995 | 0.00504 | 0.06625 | 0.06955 | 0.04637 | 0.00547 | 0.00726 | 0.00630 |
| **2** | **704.3** | 0.00188 | 0.00258 | 0.00805 | 0.00205 | 0.00222 | 0.00412 | 0.01238 | 0.01001 | 0.00585 | 0.06572 | 0.06847 | 0.04713 | 0.00561 | 0.00757 | 0.00632 |
| **2** | **704.4** | 0.00239 | 0.00360 | 0.01056 | 0.00247 | 0.00293 | 0.00211 | 0.01065 | 0.00806 | 0.00670 | 0.05426 | 0.06708 | 0.04758 | 0.00734 | 0.00920 | 0.00738 |
| **2** | **704.4** | 0.00190 | 0.00419 | 0.01025 | 0.00292 | 0.00360 | 0.00193 | 0.01147 | 0.00917 | 0.00676 | 0.05649 | 0.07004 | 0.05280 | 0.00733 | 0.00752 | 0.00735 |
| **2** | **704.4** | 0.00215 | 0.00390 | 0.01040 | 0.00270 | 0.00327 | 0.00202 | 0.01106 | 0.00861 | 0.00673 | 0.05538 | 0.06856 | 0.05019 | 0.00734 | 0.00836 | 0.00736 |
| **4** | **704.1** | 0.00105 | 0.00631 | 0.01060 | 0.00579 | 0.00589 | 0.00197 | 0.01123 | 0.00834 | 0.00766 | 0.05400 | 0.06816 | 0.04604 | 0.01179 | 0.00361 | 0.01542 |
| **4** | **704.1** | 0.00139 | 0.00531 | 0.01182 | 0.00508 | 0.00505 | 0.00213 | 0.01052 | 0.00791 | 0.00770 | 0.05010 | 0.06701 | 0.04771 | 0.00783 | 0.00561 | 0.01439 |
| **4** | **704.1** | 0.00122 | 0.00581 | 0.01121 | 0.00543 | 0.00547 | 0.00205 | 0.01088 | 0.00812 | 0.00768 | 0.05205 | 0.06758 | 0.04688 | 0.00981 | 0.00461 | 0.01491 |
| **4** | **704.2** | 0.00217 | 0.00583 | 0.01361 | 0.00512 | 0.00587 | 0.00222 | 0.01234 | 0.00988 | 0.01002 | 0.05728 | 0.06852 | 0.04973 | 0.01113 | 0.00619 | 0.01110 |
| **4** | **704.2** | 0.00156 | 0.00510 | 0.01018 | 0.00407 | 0.00478 | 0.00229 | 0.01108 | 0.00872 | 0.00731 | 0.05966 | 0.06974 | 0.04766 | 0.00770 | 0.00799 | 0.00859 |
| **4** | **704.2** | 0.00187 | 0.00546 | 0.01190 | 0.00460 | 0.00532 | 0.00226 | 0.01171 | 0.00930 | 0.00867 | 0.05847 | 0.06913 | 0.04870 | 0.00942 | 0.00709 | 0.00985 |
| **4** | **704.3** | 0.00089 | 0.00157 | 0.00475 | 0.00078 | 0.00098 | 0.00435 | 0.01273 | 0.01068 | 0.00378 | 0.07535 | 0.07564 | 0.04955 | 0.00245 | 0.00753 | 0.00292 |
| **4** | **704.3** | 0.00194 | 0.00218 | 0.00715 | 0.00156 | 0.00168 | 0.00313 | 0.01147 | 0.00896 | 0.00394 | 0.06346 | 0.07095 | 0.04812 | 0.00454 | 0.00922 | 0.00534 |
| **4** | **704.3** | 0.00141 | 0.00187 | 0.00595 | 0.00117 | 0.00133 | 0.00374 | 0.01210 | 0.00982 | 0.00386 | 0.06941 | 0.07330 | 0.04883 | 0.00349 | 0.00838 | 0.00413 |
| **4** | **704.3** | 0.00083 | 0.00151 | 0.00478 | 0.00076 | 0.00076 | 0.00424 | 0.01239 | 0.01050 | 0.00350 | 0.07423 | 0.07425 | 0.04902 | 0.00255 | 0.00728 | 0.00303 |
| **4** | **704.4** | 0.00121 | 0.00421 | 0.00875 | 0.00253 | 0.00319 | 0.00300 | 0.01039 | 0.00840 | 0.00652 | 0.06666 | 0.06987 | 0.04526 | 0.00687 | 0.00883 | 0.00774 |
| **4** | **704.4** | 0.00283 | 0.00340 | 0.01050 | 0.00254 | 0.00298 | 0.00161 | 0.01379 | 0.01101 | 0.00668 | 0.05901 | 0.07328 | 0.05616 | 0.00702 | 0.00791 | 0.00670 |
| **4** | **704.4** | 0.00202 | 0.00380 | 0.00962 | 0.00254 | 0.00309 | 0.00230 | 0.01209 | 0.00971 | 0.00660 | 0.06284 | 0.07157 | 0.05071 | 0.00695 | 0.00837 | 0.00722 |
| **7** | **704.1** | 0.00106 | 0.00172 | 0.00591 | 0.00076 | 0.00100 | 0.00157 | 0.01560 | 0.01290 | 0.00336 | 0.06720 | 0.08165 | 0.05838 | 0.00341 | 0.00698 | 0.00413 |
| **7** | **704.1** | 0.00068 | 0.00204 | 0.00533 | 0.00134 | 0.00170 | 0.00208 | 0.01247 | 0.01058 | 0.00375 | 0.06011 | 0.07154 | 0.04880 | 0.00303 | 0.00646 | 0.00473 |
| **7** | **704.1** | 0.00087 | 0.00188 | 0.00562 | 0.00105 | 0.00135 | 0.00182 | 0.01404 | 0.01174 | 0.00355 | 0.06365 | 0.07659 | 0.05359 | 0.00322 | 0.00672 | 0.00443 |
| **7** | **704.1** | 0.00091 | 0.00170 | 0.00600 | 0.00087 | 0.00095 | 0.00176 | 0.01559 | 0.01321 | 0.00385 | 0.06892 | 0.08043 | 0.05738 | 0.00310 | 0.00648 | 0.00400 |
| **7** | **704.2** | 0.00130 | 0.00259 | 0.00911 | 0.00178 | 0.00201 | 0.00236 | 0.01462 | 0.01218 | 0.00571 | 0.06631 | 0.07567 | 0.05419 | 0.00554 | 0.00819 | 0.00539 |
| **7** | **704.2** | 0.00121 | 0.00195 | 0.00705 | 0.00106 | 0.00124 | 0.00195 | 0.01380 | 0.01145 | 0.00481 | 0.06168 | 0.07174 | 0.05071 | 0.00407 | 0.01098 | 0.00417 |
| **7** | **704.2** | 0.00125 | 0.00227 | 0.00808 | 0.00142 | 0.00162 | 0.00216 | 0.01421 | 0.01182 | 0.00526 | 0.06399 | 0.07370 | 0.05245 | 0.00480 | 0.00959 | 0.00478 |
| **7** | **704.3** | 0.00120 | 0.00104 | 0.00553 | 0.00029 | 0.00051 | 0.00506 | 0.01389 | 0.01209 | 0.00474 | 0.07640 | 0.07326 | 0.04915 | 0.00207 | 0.00939 | 0.00213 |
| **7** | **704.3** | 0.00115 | 0.00141 | 0.00597 | 0.00055 | 0.00078 | 0.00419 | 0.01348 | 0.01183 | 0.00414 | 0.07408 | 0.07341 | 0.04979 | 0.00255 | 0.00787 | 0.00249 |
| **7** | **704.3** | 0.00118 | 0.00122 | 0.00575 | 0.00042 | 0.00065 | 0.00463 | 0.01368 | 0.01196 | 0.00444 | 0.07524 | 0.07333 | 0.04947 | 0.00231 | 0.00863 | 0.00231 |
| **7** | **704.3** | 0.00127 | 0.00102 | 0.00626 | 0.00028 | 0.00028 | 0.00340 | 0.01330 | 0.01117 | 0.00261 | 0.07672 | 0.07759 | 0.04994 | 0.00311 | 0.00792 | 0.00279 |
| **7** | **704.4** | 0.00188 | 0.00242 | 0.01096 | 0.00153 | 0.00159 | 0.00169 | 0.01231 | 0.00949 | 0.00569 | 0.05556 | 0.07067 | 0.04920 | 0.00644 | 0.00878 | 0.00647 |
| **7** | **704.4** | 0.00214 | 0.00262 | 0.01072 | 0.00168 | 0.00195 | 0.00175 | 0.01421 | 0.01167 | 0.00613 | 0.05968 | 0.07480 | 0.05597 | 0.00537 | 0.00908 | 0.00598 |
| **7** | **704.4** | 0.00201 | 0.00252 | 0.01084 | 0.00160 | 0.00177 | 0.00172 | 0.01326 | 0.01058 | 0.00591 | 0.05762 | 0.07274 | 0.05259 | 0.00591 | 0.00893 | 0.00622 |
| **15** | **704.1** | 0.00038 | 0.00172 | 0.00325 | 0.00082 | 0.00105 | 0.00085 | 0.01510 | 0.01323 | 0.00334 | 0.06427 | 0.07910 | 0.05681 | 0.00233 | 0.00700 | 0.00405 |
| **15** | **704.1** | 0.00055 | 0.00145 | 0.00362 | 0.00061 | 0.00083 | 0.00068 | 0.01707 | 0.01467 | 0.00268 | 0.06448 | 0.08083 | 0.06009 | 0.00252 | 0.00855 | 0.00397 |
| **15** | **704.1** | 0.00046 | 0.00158 | 0.00344 | 0.00071 | 0.00094 | 0.00076 | 0.01608 | 0.01395 | 0.00301 | 0.06437 | 0.07996 | 0.05845 | 0.00243 | 0.00778 | 0.00401 |
| **15** | **704.2** | 0.00033 | 0.00084 | 0.00224 | 0.00019 | 0.00031 | 0.00025 | 0.01850 | 0.01662 | 0.00269 | 0.07341 | 0.08875 | 0.06836 | 0.00169 | 0.01233 | 0.00264 |
| **15** | **704.2** | 0.00056 | 0.00119 | 0.00349 | 0.00042 | 0.00059 | 0.00042 | 0.01608 | 0.01416 | 0.00299 | 0.06638 | 0.08061 | 0.06343 | 0.00237 | 0.01222 | 0.00341 |
| **15** | **704.2** | 0.00044 | 0.00101 | 0.00287 | 0.00030 | 0.00045 | 0.00034 | 0.01729 | 0.01539 | 0.00284 | 0.06989 | 0.08468 | 0.06589 | 0.00203 | 0.01227 | 0.00303 |
| **15** | **704.3** | 0.00020 | 0.00105 | 0.00201 | 0.00040 | 0.00053 | 0.00251 | 0.01595 | 0.01455 | 0.00268 | 0.07899 | 0.08473 | 0.05699 | 0.00119 | 0.00722 | 0.00239 |
| **15** | **704.3** | 0.00037 | 0.00090 | 0.00283 | 0.00018 | 0.00029 | 0.00234 | 0.01579 | 0.01402 | 0.00249 | 0.07324 | 0.07950 | 0.05527 | 0.00202 | 0.00834 | 0.00264 |
| **15** | **704.3** | 0.00028 | 0.00097 | 0.00242 | 0.00029 | 0.00041 | 0.00242 | 0.01587 | 0.01428 | 0.00259 | 0.07611 | 0.08211 | 0.05613 | 0.00160 | 0.00778 | 0.00251 |
| **15** | **704.4** | 0.00201 | 0.00109 | 0.00599 | 0.00003 | 0.00027 | 0.00045 | 0.01760 | 0.01560 | 0.00386 | 0.06763 | 0.08298 | 0.07657 | 0.00222 | 0.01157 | 0.00216 |
| **15** | **704.4** | 0.00081 | 0.00176 | 0.00488 | 0.00040 | 0.00067 | 0.00036 | 0.01544 | 0.01360 | 0.00316 | 0.06136 | 0.07963 | 0.06977 | 0.00260 | 0.01039 | 0.00368 |
| **15** | **704.4** | 0.00141 | 0.00143 | 0.00544 | 0.00021 | 0.00047 | 0.00040 | 0.01652 | 0.01460 | 0.00351 | 0.06449 | 0.08131 | 0.07317 | 0.00241 | 0.01098 | 0.00292 |
